# Comprehensive analysis of serum exosome-derived lncRNAs and mRNAs from patients with rheumatoid arthritis

**DOI:** 10.1186/s13075-023-03174-9

**Published:** 2023-10-16

**Authors:** Li Xue, Biao Wang, Xueyi Li, Jianhong Zhu, Wei Wang, Fang Huang, Xiaofei Wang, Yaofeng Jin, Chaoliang Xiong, Li Tao, Ke Xu, Jing Wang, Ying Guo, Jing Xu, Xin Yang, Na Wang, Ning Gao, Yan Wang, Ke Li, Ming Li, Yan Geng

**Affiliations:** 1 Department of Clinical Laboratory, The Second Affiliated Hospital of Xi’an Jiaotong University, Xi’an, 710004 China; 2 Centre for Rheumatology and Connective Tissue Diseases, Division of Medicine, University College London, London, NW3 2PF UK; 3 Clinical Research Center for Endemic Disease of Shaanxi Province, Xi’an, 710004 China; 4 Department of Immunology and Pathogenic Biology, Health Science Center, Xi’an Jiaotong University, Xi’an, 710061 China; 5 Department of Rheumatology, The Second Affiliated Hospital of Xi’an Jiaotong University, Xi’an, 710004 China; 6 Department of Bone and Joint Surgery, The Second Affiliated Hospital of Xi’an Jiaotong University, Xi’an, 710004 China; 7 Key Laboratory of Environment and Genes Related to Diseases, Ministry of Education of China, Xi’an Jiaotong University, Xi’an, 710061 China; 8 Department of Cell Biology and Genetics, Health Science Center, Xi’an Jiaotong University, Xi’an, 710061 China; 9 Department of Pathology, The Second Affiliated Hospital of Xi’an Jiaotong University, Xi’an, 710004 China; 10 Department of Joint Surgery, Xi’an Hong Hui Hospital, Xi’an Jiaotong University Health Science Center, Xi’an, 710049 China; 11 Department of Rheumatology, The First Affiliated Hospital of Xi’an Jiaotong University, Xi’an, 710061 China; 12 National Local Joint Engineering Research Centre of Biodiagnostics and Biotherapy, The Second Affiliated Hospital of Xi’an Jiaotong University, Xi’an, 710004 China; 13 Department of Biochemistry and Molecular Biology, Xi’an Jiaotong University Health Science Center, Xi’an, 710061 China; 14 Core Research Laboratory, The Second Affiliated Hospital of Xi’an Jiaotong University, Xi’an, 710004 China; 15 Department of Emergency, The First Affiliated Hospital of Xi’an Jiaotong University, Xi’an, 710061 China

**Keywords:** Rheumatoid arthritis, Serum exosomes, lncRNA, mRNAs, RNA-sequencing

## Abstract

**Background:**

Serum exosomes play important roles in intercellular communication and are promising biomarkers of several autoimmune diseases. However, the biological functions and potential clinical importance of long non-coding RNAs (lncRNAs) and mRNAs from serum exosomes in rheumatoid arthritis (RA) have not yet been studied.

**Methods:**

Serum exosomal lncRNAs and mRNAs were isolated from patients with RA and osteoarthritis (OA) and healthy controls. The differentially expressed lncRNAs (DE-lncRNAs) and mRNA profiles in the serum exosomes of patients with RA were analysed using high-throughput sequencing, and their functions were predicted using Gene Ontologyenrichment, Kyoto Encyclopedia of Genes and Genomes pathway, and gene set enrichment analysis. We constructed a DE-lncRNA-mRNA network and a protein–protein interaction network of differentially expressed mRNAs (DE-mRNAs) in RA using the Cytoscape software. The expression of several candidate a DE-lncRNAs and DE-mRNAs in the serum of patients with RA, patients with OA, and healthy controls was confirmed by qRT-PCR. We assessed the diagnostic ability of DE-lncRNAs and DE-mRNAs in patients with RA using receiver operating characteristic analysis. Furthermore, we analysed the characteristics of immune cell infiltration in RA by digital cytometry using the CIBERSORT algorithm and determined the correlation between immune cells and several DE-lncRNAs or DE-mRNAs in RA.

**Results:**

The profiles of serum exosomal lncRNAs and mRNAs in patients with RA were different from those in healthy controls and patients with OA. The functions of both DE-lncRNAs and DE-mRNAs in RA are associated with the immune response and cellular metabolic processes. The RT-PCR results show that NONHSAT193357.1, CCL5, and MPIG6B were downregulated in patients with RA. The combination of three DE-mRNAs, CCL5, MPIG6B, and PFKP, had an area under the curve of 0.845 for differentiating RA from OA. Digital cytometry using the CIBERSORT algorithm showed that the neutrophil counts were higher in patients with RA than those in healthy controls and patients with OA.

**Conclusions:**

These findings help to elucidate the role of serum exosomal lncRNAs and mRNAs in the specific mechanisms underlying RA.

**Supplementary Information:**

The online version contains supplementary material available at 10.1186/s13075-023-03174-9.

## Introduction

Rheumatoid arthritis (RA) is a chronic autoimmune disease affecting approximately 1% of the global population [1]. Although great progress has been made in the diagnosis and treatment of RA in recent years, damage to the articular cartilage and bone and long-term disability in patients are still common [2]. According to the criteria recommended by the European League Against Rheumatism in 2010, rheumatoid factor and anti-cyclic citrullinated peptide are the currently available diagnostic indices for RA. However, the sensitivity and specificity of these two markers were not optimal for RA [3]. Therefore, it is essential to identify novel and effective diagnostic and prognostic markers to improve RA diagnosis.

Increasing amounts of evidence suggest that circulating extracellular vesicles are promising biomarkers for the diagnosis, prognosis, and therapeutic evaluation of patients with autoimmune diseases [4–6]. Exosomes usually contain multiple bioactive molecules, such as RNAs, including mRNA, microRNAs, and long non-coding RNAs. They also contain proteins that play important roles in intercellular communication and signalling by transferring their internal cargo to recipient cells [7]. For example, a previous study reported that miR-548a3p was significantly reduced in the serum exosomes of patients with RA and that there was a negative correlation between serum exosomal miR-548a-3p and key laboratory indicators associated with disease activity in RA [8]. Another study indicated that serum exomiR-451a and exomiR-25-3p may provide a novel serum biomarker panel for early clinical diagnosis of RA [9]. Although these findings indicate that microRNAs (miRNAs) in serum exosomes could potentially be used as diagnostic biomarkers for RA, a comprehensive analysis of long non-coding RNAs and mRNAs and their interactions in the serum exosomes of patients with RA has not yet been conducted.

Long non-coding RNAs (lncRNAs) are more than 200 nucleotides in length with no apparent protein-coding role. lncRNA expression profiles are important markers and therapeutic targets for autoimmune diseases, such as RA and systemic lupus erythematosus [10–14]. lncRNAs are also involved in post-transcriptional regulation by interacting with miRNAs, mRNAs, and proteins [15, 16]. A previous study revealed that Hotair lncRNA levels were elevated in the exosomes of peripheral blood mononuclear cells (PBMCs) and serum from patients with RA compared to normal controls, and Hotair has been functionally implicated in the migration of active macrophages [17]. Recent evidence has also shown that the lncRNA NEAT1 derived from PBMC exosomes contributes to RA development via the miR-23a/MDM2/SIRT6 axis [18] and that serum-derived exosomes containing NEAT1 may promote RA occurrence by regulating the miR-144-3p/ROCK2 axis [19]. Despite these findings indicating the participation of several exosome-derived lncRNAs in RA pathogenesis, lncRNA expression profiles in the serum of patients have not been fully explored.

This study aimed to determine whether serum-derived exosomal lncRNAs and mRNA function in RA and could potentially be used as diagnostic biomarkers. First, we purified exosomal lncRNAs and mRNAs from patients with RA and osteoarthritis (OA) and healthy controls. We conducted RNA sequencing to obtain expression profiles and performed functional analyses of differentially expressed lncRNAs (DE-lncRNAs) and differentially expressed mRNAs (DE-mRNAs) in serum-derived exosomes from patients with RA. Second, we analysed the biological functions of these DE-lncRNAs and DE-mRNAs, and constructed a DE-lncRNA-mRNA network and a protein–protein interaction (PPI) network of DE-mRNAs in patients with RA using bioinformatics. Third, we validated the expression of several candidate DE-lncRNAs and DE-mRNAs using qRT-PCR and assessed the diagnostic utility of DE-lncRNAs and DE-mRNAs in patients with RA using receiver operating characteristic (ROC) analysis. Finally, we explored the characteristics of immune infiltration in RA using the CIBERSORT algorithm and analysed the correlation between immune cells and DE-lncRNAs or DE-mRNAs in patients with RA.

## Methods

### Study population and sample collection

The study population included 19 patients with RA, 19 patients with OA, and 19 healthy controls. Patients with RA were admitted to the Department of Rheumatology and Immunology of the Second Affiliated Hospital of Xi’an Jiaotong University between September 2020 and September 2021 and fulfilled the criteria of the 2010 American College of Rheumatology. Age-and sex-matched healthy participants and patients with OA were recruited from the Health Examination Center and Department of Orthopedics of the Second Affiliated Hospital of Xi’an Jiaotong University, respectively. Serum exosomes from four patients with RA, four patients with OA, and four healthy controls were subjected to RNA sequencing. Another cohort of 15 patients with RA, 15 with OA, and 15 healthy controls was subjected to qRT-PCR verification. This study was approved by the Research Committee of Human Investigation of the Xi’an Jiaotong University Health Science Center, and all participants provided written informed consent.

### Exosome isolation from serum

Exosomes were isolated from the serum samples by differential centrifugation, which was optimised according to a previously described protocol [4]. Serum samples were centrifuged at 3000 × g for 15 min to remove cell debris. Following this, the supernatant was diluted with seven-fold the volume of phosphate-buffered saline (PBS), centrifuged at 13,000 × g for 30 min, and processed through a 0.22-μm filter to remove large particles. The supernatant was ultracentrifuged using a P50A72-986 rotor (CP100NX; Hitachi, Brea, CA, USA) at 100,000 × g, 4 °C for 2 h to pellet the exosomes. The pellet was resuspended in PBS and centrifuged again at 100,000 × g 4 °C for 2 h. After washing, the exosome pellet was resuspended in 100 μl PBS and stored at − 80 °C until use.

### Nanoparticle tracking analysis (NTA)

Suspensions of prepared exosomes with concentrations ranging from 1 × 10^7^ to 1 × 10^9^ /ml were examined using ZetaView PMX 110 (Particle Metrix, Meerbusch, Germany) to determine the size and quantity of the exosomes. Particle movement was analysed using NTA software (ZetaView 8.02.28).

### Transmission electron microscopy (TEM)

The morphology of the exosomes was examined using TEM. Briefly, 10 μl exosome solution was placed on a copper mesh and incubated at room temperature for 1 min. The residual fluid was removed using filter paper. The exosomes were then negatively stained with uranyl acetate solution for 1 min before the samples were dried for 2 min. The copper mesh was loaded onto the sample holder of a transmission electron microscope (H-7650, Hitachi Ltd., Tokyo, Japan), and images of the exosomes were captured.

### Western blot analysis

Exosomal protein concentration was evaluated using a BCA kit (Thermo Fisher Scientific, Waltham, MA, USA) according to the manufacturer’s instructions. First, 50 μg of each protein sample was separated using 10% SDS–polyacrylamide gel electrophoresis before the proteins were transferred onto polyvinylidene fluoride membranes (Millipore, Billerica, MA, USA). These membranes were blocked with 5% non-fat milk TBST for 1 h at room temperature then incubated with the primary antibodies anti-CD9 (Cell Signalling Technology), anti-Tsg101 (Abcam), anti-HSP70 (Cell Signalling Technology), and anti-Calnexin (Abcam) overnight at 4 °C. The membranes were washed thrice with TBST for 10 min, incubated with an HRP-conjugated secondary antibody (Cell Signalling Technology) for 2 h at room temperature, and washed with TBST. Finally, the membranes were visualised using a detection reagent (chemiluminescent HRP substrate; Millipore).

### RNA sequencing of serum exosomes

Total RNA isolated from serum exosomes was purified using an RNA Clean XP Kit (Beckman Coulter, Brea, CA, UA). RNA quality and concentration were assessed using a NanoDrop ND-1000 spectrophotometer (Thermo Fisher Scientific). RNA integrity was determined using an Agilent 2200 TapeStation (Agilent Technologies), and samples with an RNA integrity number > 7.0 were considered acceptable for further sequencing. Total RNA samples were fragmented to approximately 200 bp. The NEBNext Ultra RNA Library Prep Kit for Illumina (NEB) was used to prepare the RNA-seq libraries. The quality of each library was controlled using the Agilent 2200 TapeStation (Agilent Technologies). Clustering of index-coded samples was performed using the HiSeq Rapid PE Cluster Kit V2 (Illumina). After cluster generation, libraries were sequenced using the Illumina HiSeq 3000 platform.

### Differential expression analysis of lncRNA and mRNA

Quantification of mRNA expression levels and differential expression analyses were conducted on the mapped reads. Differential expression analysis of mRNAs and lncRNAs between different groups was performed using the Mann–Whitney *U* test with a cutoff FPKM > 5, *p*-value < 0.05, and fold change > 2.0. Heat maps and volcano diagrams were generated to visualise the DE-lncRNAs and DE-mRNAs using the R packages “pheatmap” and “ggplot2”. Principal component analysis (PCA) and Venn analysis were performed using FactoMineR and the Vennerable Venn Diagram in R packages, respectively.

### RNA isolation and qRT-PCR

Total RNA was extracted from the exosomes using the miRNeasy Serum/Plasma Kit (Qiagen, Boston, MA, USA) following the manufacturer’s protocol. Total RNA was then used for reverse transcription and cDNA was synthesised using PrimeScript™ RT reagent Kit (TAKARA). The abundance of target genes was detected using TaqMan probes and qRT-PCR. The primer and probe sequences are listed in Table 1. The comparative CT (2^−ΔΔCT^) method was used to obtain the fold change in relative expression levels of candidate lncRNAs and mRNAs. All experiments were performed in triplicate.

**Table 1:** Primer sequences

	**Primer name**	**Primer sequence (5′-3′)**
Forward primer	GAPDH-F	TCAGCCGCATCTTCTTTTGC
Reverse primer	GAPDH-R	GCCCAATACGACCAAATCCG
Probe	GAPDH-P	TCGCCAGCCGAGCCACATC
Forward primer	MIR3945HG-202-F	CCACCACTTTGGGAGGC
Reverse primer	MIR3945HG-202-R	GGAGTCTTGCTGTATTGCCTA
Probe	MIR3945HG-202-P	TGAGCCCAGGAGTTGGAGACC
Forward primer	NONHSAT193357.1-F	CGAGTGAACAGGGAAGAGC
Reverse primer	NONHSAT193357.1-R	GGAGTTTACCACCCGCTTT
Probe	NONHSAT193357.1-P	AGTCGGGTTGCTTGGGAATGC
Forward primer	NONHSAT190641.1-F	CGCTAAACCATTCGTAGACGA
Reverse primer	NONHSAT190641.1-R	AATAGATCGCAGCGAGGGA
Probe	NONHSAT190641.1-P	CTTCTGGGTCGGGGTTTCGTAC
Forward primer	CCL5-F	CATTGCTACTGCCCTCTGC
Reverse primer	CCL5-R	ACACTTGGCGGTTCTTTCG
Probe	CCL5-P	CACACCCTGCTGCTTTGCCTAC
Forward primer	PFKP-F	CGGCTACTGTGGCTACCT
Reverse primer	PFKP-R	CGCCTTTGCCCTCTTCTG
Probe	PFKP-P	CGTGGAGCACCTGACGGAGAA
Forward primer	MPIG6B-F	TGTCCAAAGGACGCCGA
Reverse primer	MPIG6B-R	CCCAGTCCGAGCACCAA
Probe	MPIG6B-P	ACGCTCCCTGGACTCTGGTATC

### Functional enrichment analysis

The top Gene Ontology (GO) terms and Kyoto Encyclopedia of Genes and Genomes (KEGG) signalling pathways enriched in DE-lncRNAs and DE-mRNAs were identified and visualised using the Cluster Profiler package. Gene set enrichment analysis (GSEA) was conducted using the gene pathways extracted from GO and KEGG. The rankings and differences were verified by calculating the statistical significance of the normalised enrichment score using the nominal *p*-value and FDR *q*-value. The cut-offs for the *p*-value and FDR *q*-value were set at 0.05 and 0.10.

### Construction of RNA interaction and PPI networks

An lncRNA-mRNA interaction network was constructed based on the binding of the five selected lncRNAs to their target mRNAs. The five selected lncRNAs were specifically expressed in RA compared to the controls and OA. Additionally, we used the STRING database to predict the association between the DE-mRNA-coding proteins. An lncRNA-mRNA network and a PPI network were visualised using the Cytoscape software.

### Immune cell infiltration analysis

CIBERSORT, a bioinformatics algorithm, was used to analyse the different types of infiltrating immune cells from the gene expression profiles of the three groups (RA, OA, and healthy controls). R package “xCell” was used to perform analysis on the scoring matrix of the gene expression profiles of three different groups. A heat map of different cell types was constructed using the R package “pheatmap”. Correlation analysis between infiltrating immune cells and DE-lncRNAs or DE-mRNAs was performed using the R package “corrplot”.

### Statistical analysis

Statistical analysis was performed using GraphPad Prism (version 8.0; GraphPad Software, Inc., CA, USA) and SPSS (version 20.0; IBM, Armonk, NY, USA). The R software (v. 3.5.3) was used to construct the ROC curves, and the area under the ROC curve (AUC) was calculated using a stepwise logistic regression algorithm. The qRT-PCR results were compared using one-way analysis of variance combined with Tukey’s multiple comparison test. *p* < 0.05 was considered statistically significant.

## Results

### Characteristics of serum exosomes

**Fig. 1:**
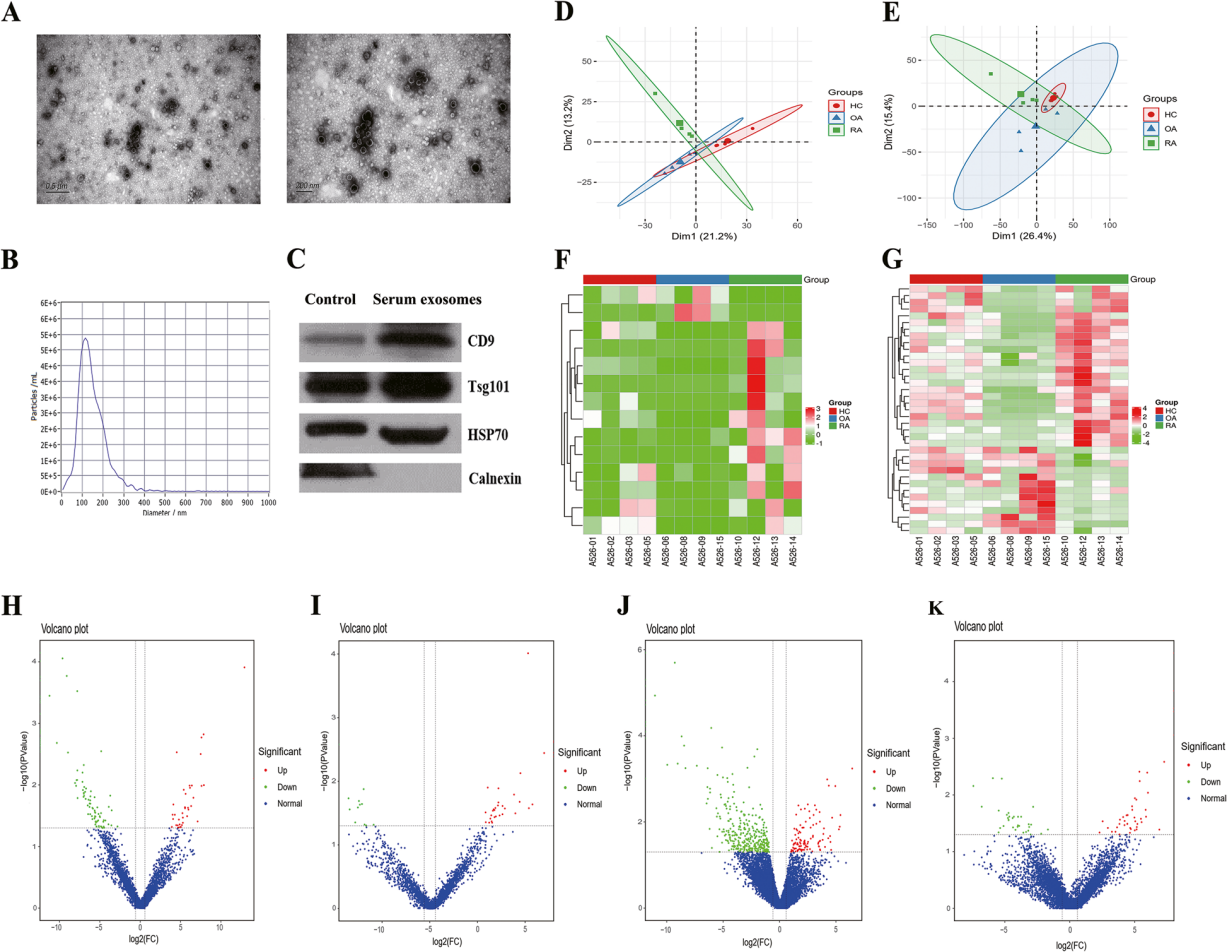
Analysis of differentially expressed exosomal lncRNAs and mRNAs in patients with RA. **A**–**C** Identification of exosomes isolated from human serum by transmission electron microscopy, nanoparticle tracking analysis, and western blotting. **D**–**E** Principal component analysis of lncRNAs (**D**) and mRNAs (**E**) from all samples. **F**–**G** Heat maps of the hierarchical clustering analysis of overall DE-lncRNAs (**F**) and DE-mRNAs (**G**) in serum exosomes from patients with RA. Cut-off values: *p* < 0.05 and fold change > 2. **H**–**K** Volcano plot of DE-lncRNAs (**H**, **I**) and DE-mRNAs (**G**, **K**) in serum exosomes from patients with RA. **H** and **G** represent the comparison between patients with RA and healthy controls, while **I** and **K** represent the comparison between patients with RA and patients with OA. DE-lncRNAs, differentially expressed lncRNAs; DE-mRNAs, differentially expressed mRNAs; RA, rheumatoid arthritis; OA, osteoarthritis

To determine whether the particles isolated from the serum were exosomes, we characterised the vesicles using TEM, NTA, and protein analysis. Spherical vesicles with diameters ranging from 30 to 200 nm were observed using TEM (Fig. 1A). NTA showed that most of the isolated serum-derived vesicles were between 30 and 150 nm in diameter (Fig. 1B), which is compatible with the conventional size range of exosomes. Western blot analysis validated the expression of exosome-specific proteins CD9, Tsg101, and HSP70 (Fig. 1C).

### Identification of DE-lncRNAs and DE-mRNAs in serum exosomes of patients with RA

Exploratory analysis of DE-lncRNAs and DE-mRNAs from serum exosomes showed significant differences in expression patterns between patients with RA and healthy controls and between patients with RA and OA. PCA was performed on all samples, and PCA of lncRNA and mRNA expression showed divergent trends in signal intensity distributions, indicating altered lncRNA and mRNA expression profiles in serum exosomes of patients with RA compared with those of healthy controls and patients with OA (Fig. 1D, E). Hierarchical clustering and volcano plot analysis revealed that the expression profiles of exosomal DE-lncRNAs and DE-mRNAs could distinguish patients with RA from healthy controls and patients with OA (Fig. 1F–K).

### GO analysis

**Fig. 2:**
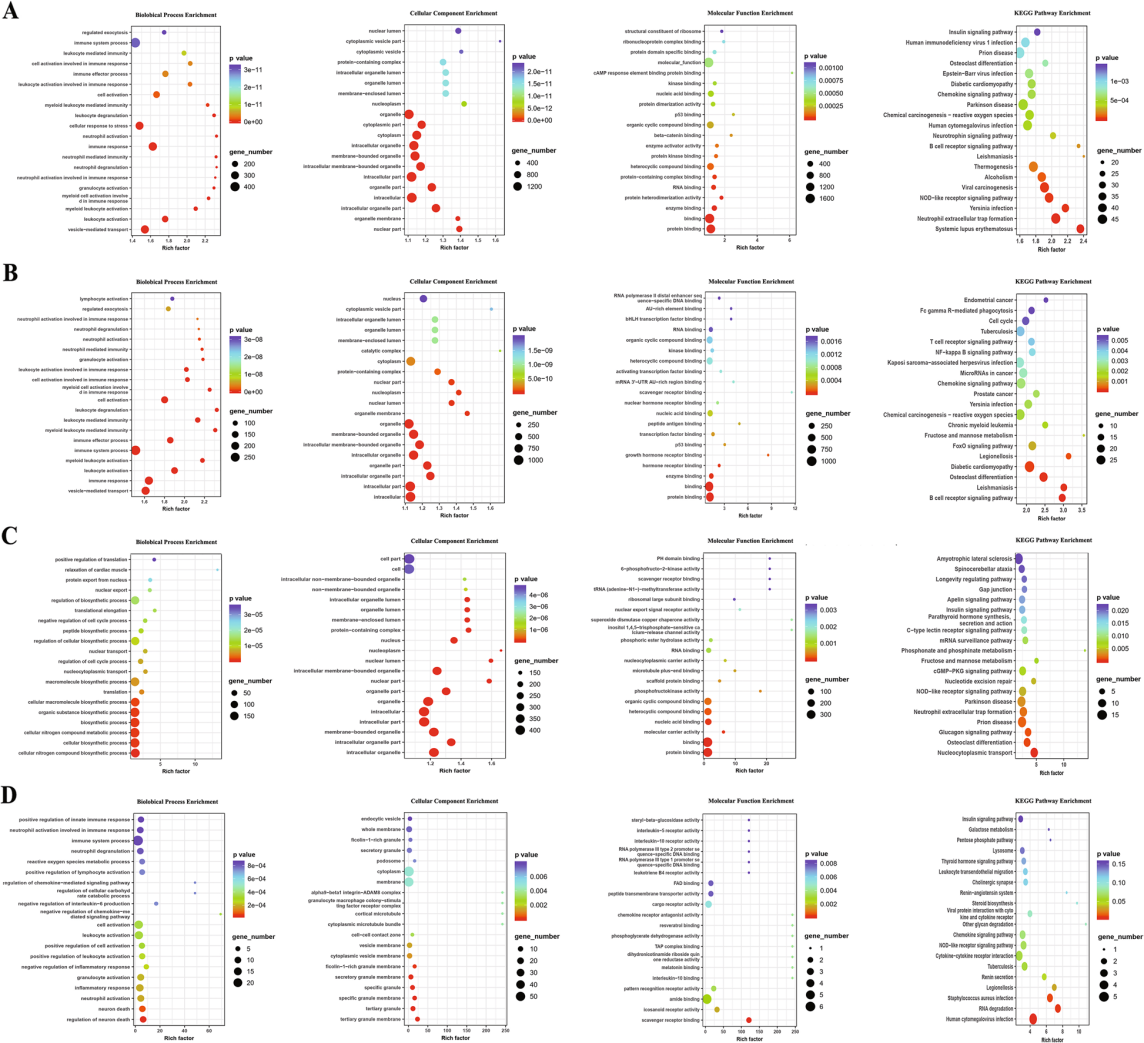
Functional analyses of significantly expressed lncRNAs and mRNAs in serum exosomes of patients with RA. **A**–**B** GO analysis and KEGG pathway enrichment of the DE-lncRNAs in patients with RA compared with healthy controls (**A**) and patients with OA (**B**). **C**–**D** GO analysis and KEGG pathway enrichment of the DE-mRNAs in patients with RA compared with healthy controls (**C**) and patients with OA (**D**) G O, Gene Ontology; KEGG, Kyoto Encyclopedia of Genes and Genomes; DE-lncRNAs, differentially expressed lncRNAs; DE-mRNAs, differentially expressed mRNAs; RA, rheumatoid arthritis; OA, osteoarthritis

To determine the potential molecular functions of the DE-lncRNAs and DE-mRNAs discovered in the serum exosomes of patients with RA, GO enrichment and KEGG pathway analyses were performed to evaluate the biological functions and cellular signalling pathways of their target genes. The enriched GO terms were grouped into three categories: biological processes, cellular components, and molecular functions. The top 20 GO terms and KEGG signalling pathways of the DE-lncRNAs between RA and healthy controls are shown in Fig. 2A, whereas those of the DE-lncRNAs between RA and OA are shown in Fig. 2B. The results reveal that the most significantly enriched GO terms of DE-lncRNAs between patients with RA and healthy controls were associated with leukocyte activation involved in the immune response, neutrophil activation, and myeloid leukocyte-mediated immunity. The most significantly enriched KEGG pathways of these DE-lncRNAs between patients with RA and healthy controls were related to the B cell receptor signalling, chemokine signalling, NOD receptor signalling, and insulin signalling pathways, as well as neutrophil extracellular trap formation and osteoclast differentiation (Fig. 2A). GO analysis showed that the target genes of the DE-lncRNAs between RA and OA were mostly enriched in lymphocyte activation, neutrophil-mediated immunity, and leukocyte activation involved in the immune response. The most significantly enriched KEGG pathways of these DE-lncRNAs between RA and OA were associated with the T cell receptor signalling, NF − kappa B signalling, chemokine signalling, FoxO signalling, and B cell receptor signalling pathways, as well as osteoclast differentiation (Fig. 2B).

Functional enrichment analysis was also performed on DE-mRNA in RA. The top 20 GO and KEGG terms between the RA and healthy controls and between the RA and OA groups are shown in Figs. 2C and D, respectively. GO analysis showed that the DE-mRNA in patients with RA and healthy controls were mostly enriched in the regulation of biosynthetic processes, negative regulation of cell cycle processes, and macromolecule biosynthetic processes. The most significantly enriched KEGG pathways of these DE-mRNAs between patients with RA and healthy controls were associated with cGMP − PKG signalling, the NOD receptor signalling pathway, neutrophil extracellular trap formation, and osteoclast differentiation (Fig. 2C). The most significantly enriched GO terms of DE-mRNAs between RA and OA were associated with the positive regulation of innate immune response, neutrophil activation involved in immune response, the reactive oxygen species metabolic process, positive regulation of lymphocyte activation, regulation of chemokine-mediated signalling pathways, and positive regulation of leukocyte activation. The most significantly enriched KEGG pathways of the DE-mRNAs between RA and OA were associated with leukocyte transendothelial migration, chemokine signalling, NOD receptor signalling, and cytokine-cytokine receptor interactions (Fig. 2D).

**Fig. 3:**
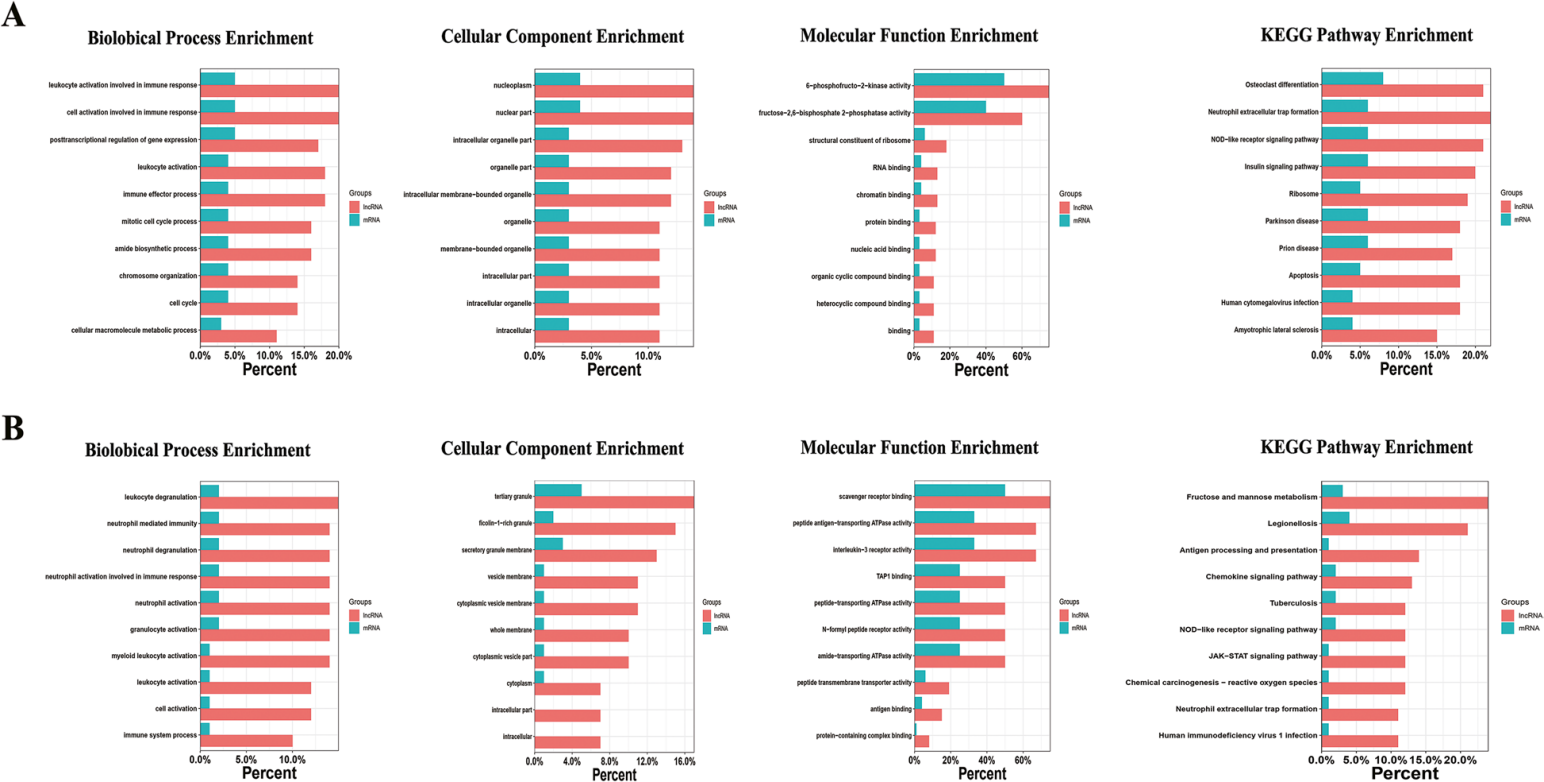
Analysis of common functions of both differentially expressed lncRNAs and mRNAs in serum exosomes of patients with RA. GO analysis and KEGG pathway enrichment of both DE-lncRNAs and DE-mRNAs in patients with RA compared with healthy controls (**A**) and patients with OA (**B**). GO, Gene Ontology; KEGG, Kyoto Encyclopedia of Genes and Genomes; DE-lncRNAs, differentially expressed lncRNAs; DE-mRNAs, differentially expressed mRNAs; RA, rheumatoid arthritis; OA, osteoarthritis

In this study, GO analysis and KEGG pathway enrichment were also used to investigate the common functions of both DE-lncRNAs and DE-mRNAs in serum exosomes of patients with RA compared to healthy controls and patients with OA. The top 10 GO terms and KEGG signalling pathways between patients with RA and healthy controls and between RA and patients with OA are shown in Figs. 3A and B, respectively. The results reveal that the most significantly enriched GO terms for both the DE-lncRNAs and DE-mRNAs between RA and OA were related to leukocyte degranulation, neutrophil activation involved in the immune response, and myeloid leukocyte activation. The chemokine signalling, NOD receptor signalling, and JAK-STAT signalling pathways, as well as neutrophil extracellular trap formation, were identified as the most significantly enriched KEGG pathways for both DE-lncRNAs and DE-mRNAs between RA and OA.

### GSEA analysis of DE-mRNAs

GSEA revealed that the KEGG pathways of these upregulated mRNAs in RA were related to cytokine-cytokine receptor interaction, NOD-like receptor signalling, osteoclast differentiation, and TNF signalling pathways in comparison with OA. For those downregulated mRNAs in RA relative to OA, the KEGG pathway was related to pyruvate metabolism; tryptophan metabolism; propanoate metabolism; glycine, serine, and threonine metabolism; amino acid biosynthesis; glycolysis/gluconeogenesis; oxidative phosphorylation; and protein processing in the endoplasmic reticulum (Supplementary Fig. S1D).

### LncRNA-mRNA network results

**Fig. 4:**
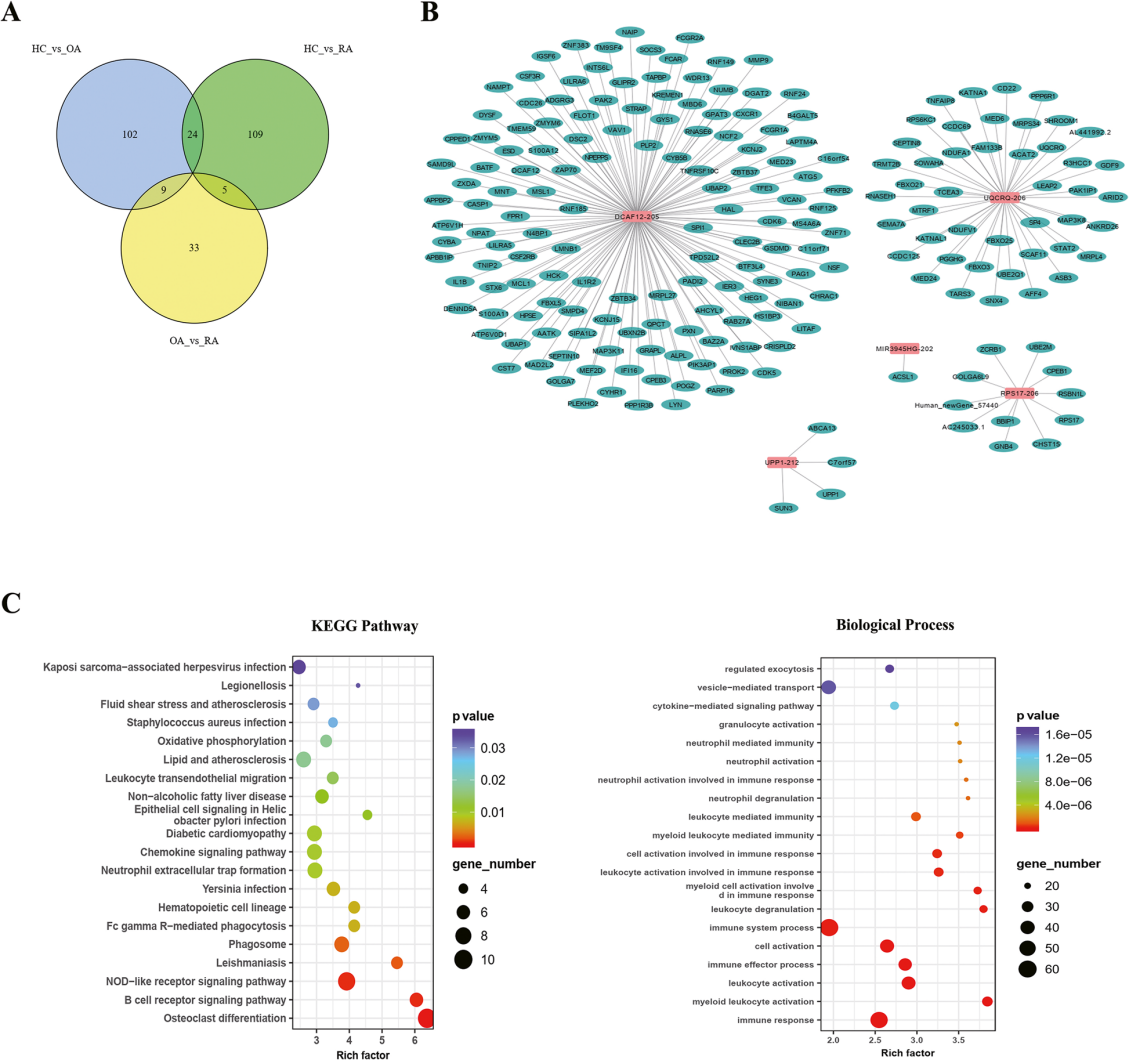
LncRNA-mRNA interaction network and functional enrichment of lncRNA-target mRNAs for serum-derived exosomes of patients with RA. **A** Venn diagram of the DE-lncRNAs of healthy controls-versus-RA, healthy controls-versus-OA, and OA-versus-RA. **B** Interaction network of five DE-lncRNAs and their target mRNAs in patients with RA. **C** KEGG pathway enrichment and biological process terms of the five DE-lncRNAs-target genes. DE-lncRNAs, differentially expressed lncRNAs; RA, rheumatoid arthritis; OA, osteoarthritis

To reveal the regulatory role of lncRNAs in protein-coding genes associated with RA pathogenesis, an lncRNA-mRNA network consisting of five lncRNAs (differentially expressed in serum exosomes of patients with RA compared with healthy controls and patients with OA; Fig. 4A) and target mRNAs was constructed (Fig. 4B). The enriched functional KEGG terms for these target mRNAs in the network were oxidative phosphorylation, leukocyte transendothelial migration, neutrophil extracellular trap formation, and the chemokine signalling, NOD − like receptor signalling, and B cell receptor signalling pathways (Fig. 4C). In addition, the most relevant biological processes related to RA, including the cytokine-mediated signalling pathway, neutrophil activation involved in the immune response, and leukocyte-mediated immunity, were enriched for target mRNAs in the network (Fig. 4D).

### PPI network results

A PPI network using the serum exosomal DE-mRNAs in RA compared with OA was constructed to investigate the functional role of these protein interactions in RA pathogenesis. As shown in Supplementary Fig. S2, the PPI network was divided into three clusters. Cluster 2 proteins (green symbols) were involved in leukocyte activation, neutrophil activation, innate immune response, cellular response to cytokine stimulus, and leukocyte migration. There were no significant differences in the functional analysis of the enriched genes of cluster 1 and 3 proteins.

### Validation of candidate lncRNAs and mRNAs with qRT-PCR

**Fig. 5:**
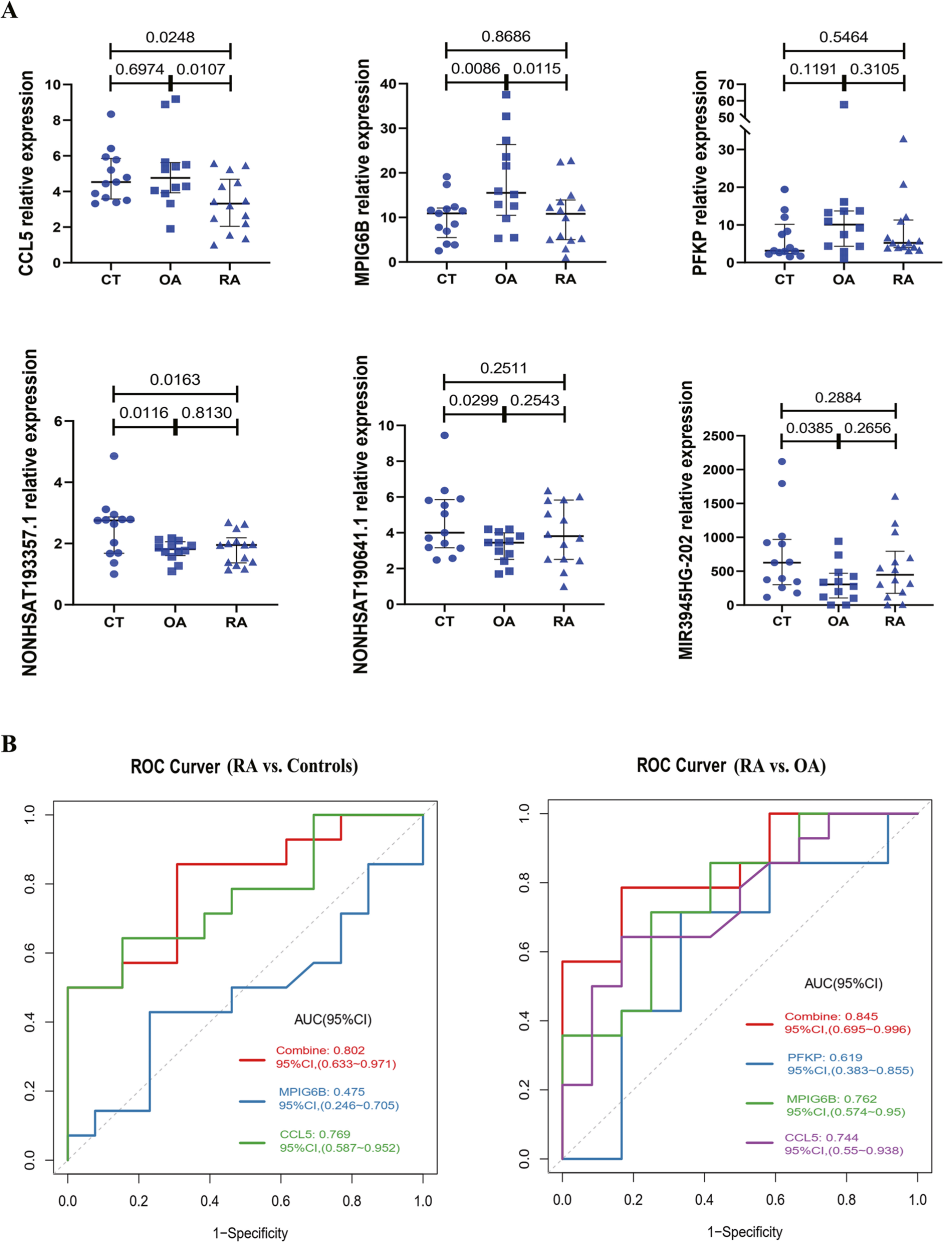
QRT-PCR validation and ROC analysis of selected DE-lncRNAs and DE-mRNAs in serum exosomes. **A** Validation of DE-lncRNAs (MIR3945HG-202, NONHSAT193357.1, and NONHSAT190641.1) and DE-mRNAs (CCL5, PFKP, and MPIG6B) in serum exosomes of patients with RA, healthy controls, and patients with OA by qRT-PCR. **B** ROC curves of the DE-lncRNAs and DE-mRNAs in differentiating patients with RA from healthy controls or patients with OA. ROC, receiver operating characteristic; DE-lncRNAs, differentially expressed lncRNAs; DE-mRNAs, differentially expressed mRNAs; RA, rheumatoid arthritis; OA, osteoarthritis

To verify the results of lncRNA and mRNA sequencing, we used qRT-PCR to detect the expression of three lncRNAs (MIR3945HG-202, NONHSAT193357.1, and NONHSAT190641.1) and three mRNAs (CCL5, PFKP, and MPIG6B) selected from serum exosomes in an independent validation cohort of patients with RA, healthy controls, and patients with OA. As shown in Fig. 5A, the relative expression level of CCL5 was significantly downregulated in patients with RA compared to that in healthy controls and patients with OA. The relative expression level of MPIG6B was significantly upregulated in patients with OA compared to that in healthy controls and patients with RA. These results were similar to those of the sequence profiling. For lncRNAs, the relative expression level of NONHSAT193357.1 was significantly downregulated in patients with RA and OA compared with healthy controls.

### ROC curve analysis

To further evaluate the diagnostic efficiency of DE-lncRNAs and DE-mRNAs in serum exosomes to distinguish RA patients from healthy controls or OA patients, we performed ROC curve analysis of several DE-lncRNAs or DE-mRNAs which had been validated by qRT-PCR. In patients with RA versus healthy controls, CCL5 and PFKP had AUCs of 0.77 and 0.68, respectively (Table 2). As shown in Table 2, CCL5 had high sensitivity, while MPIG6B had high specificity for distinguishing patients with RA from healthy controls. Therefore, we evaluated the diagnostic performance of the combination of CCL5 and MPIG6B, which had an AUC of 0.802 (Fig. 5B). For patients with RA versus OA, the AUCs for CCL5, MPIG6B, and PFKP were 0.744, 0.762, and 0.619, respectively (Table 3). The combination of CCL5, MPIG6B, and PFKP had an AUC of 0.845 (Fig. 5B).

**Table 2:** The diagnostic value of CCL5, PFKP, MPIG6B, and NONHSAT190641.1 for distinguishing RA from healthy controls

Parameters	AUC	*p* value	Specificity (%)	Sensitivity (%)	NPV (%)	AC (%)
CCL5	0.769	0.0295	84.62	64.29	68.75	74.07
PFKP	0.681	0.3950	53.85	100	100	77.78
MPIG6B	0.475	0.8260	76.92	28.57	50	51.85
NONHSAT190641.1	0.593	0.3105	92.31	35.71	57.14	62.96

**Table 3:** The diagnostic value of CCL5, PFKP, MPIG6B, and NONHSAT190641.1 for distinguishing RA from OA

Parameters	AUC	*P* value	Specificity (%)	Sensitivity (%)	NPV (%)	AC (%)
CCL5	0.744	0.04209	83.33	64.29	66.67	73.08
PFKP	0.619	0.3935	66.67	71.43	66.67	69.23
MPIG6B	0.762	0.04645	58.33	85.71	77.78	73.08
NONHSAT190641.1	0.619	0.1948	1	42.86	60.00	69.23

*AUC*, area under curve, *NPV* negative predictive value, *AC* accuracy

### Immune infiltration analysis

**Fig. 6:**
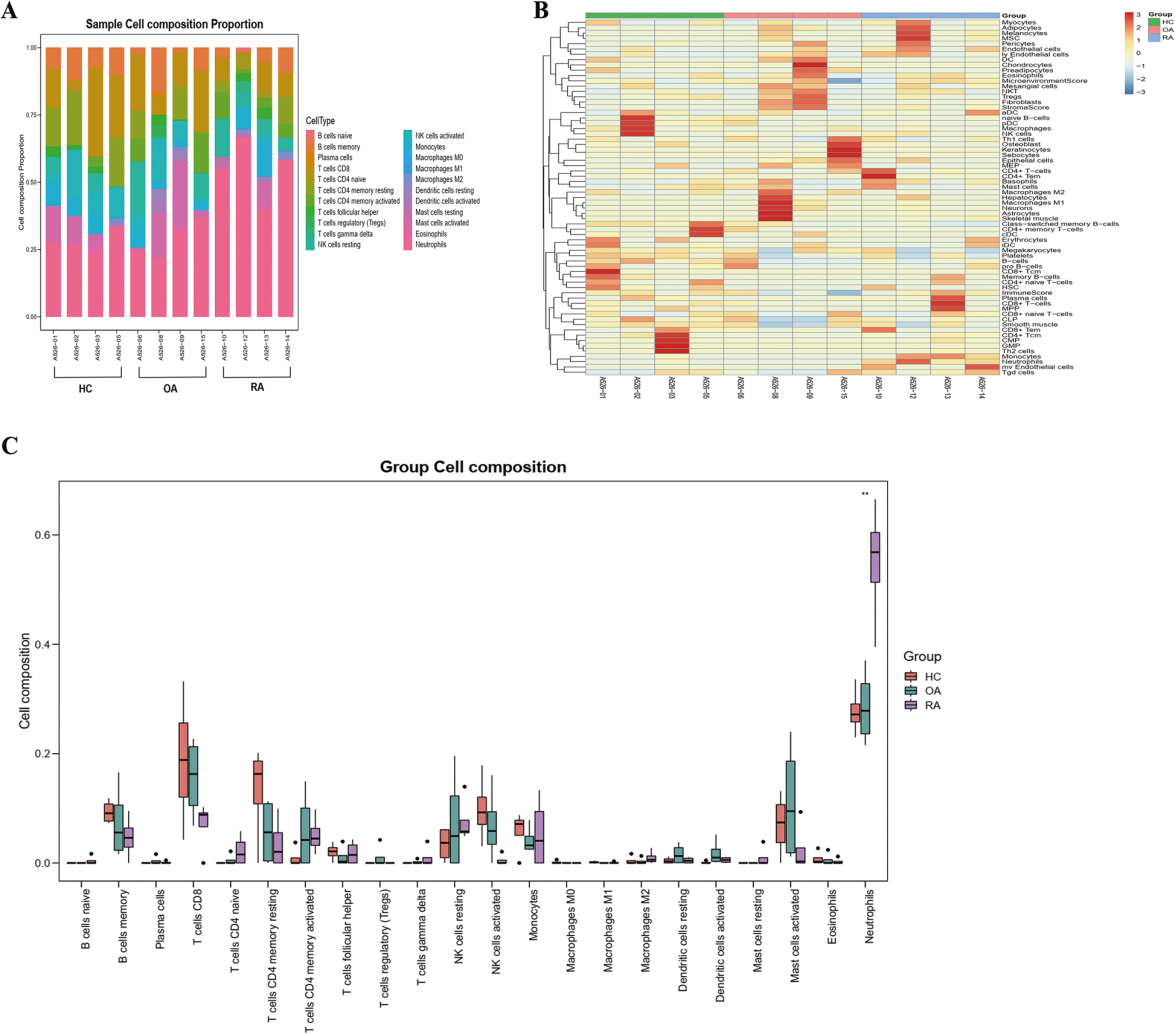
The landscape of immune infiltration in three groups including patients with RA, healthy controls, and patients with OA. **A** The relative percentage of 22 types of immune cell. CIBERSORT was adopted to analyse the different types of infiltrating immune cells from the gene expression profiles of three groups. **B** Heat map of different cell types. R package “xCell” was used to perform analyses on the scoring matrix of the gene expression profiles of three groups. **C** Difference of immune infiltration between the three groups. *p* < 0.05 was considered statistically significant; RA, rheumatoid arthritis; OA, osteoarthritis

**Fig. 7:**
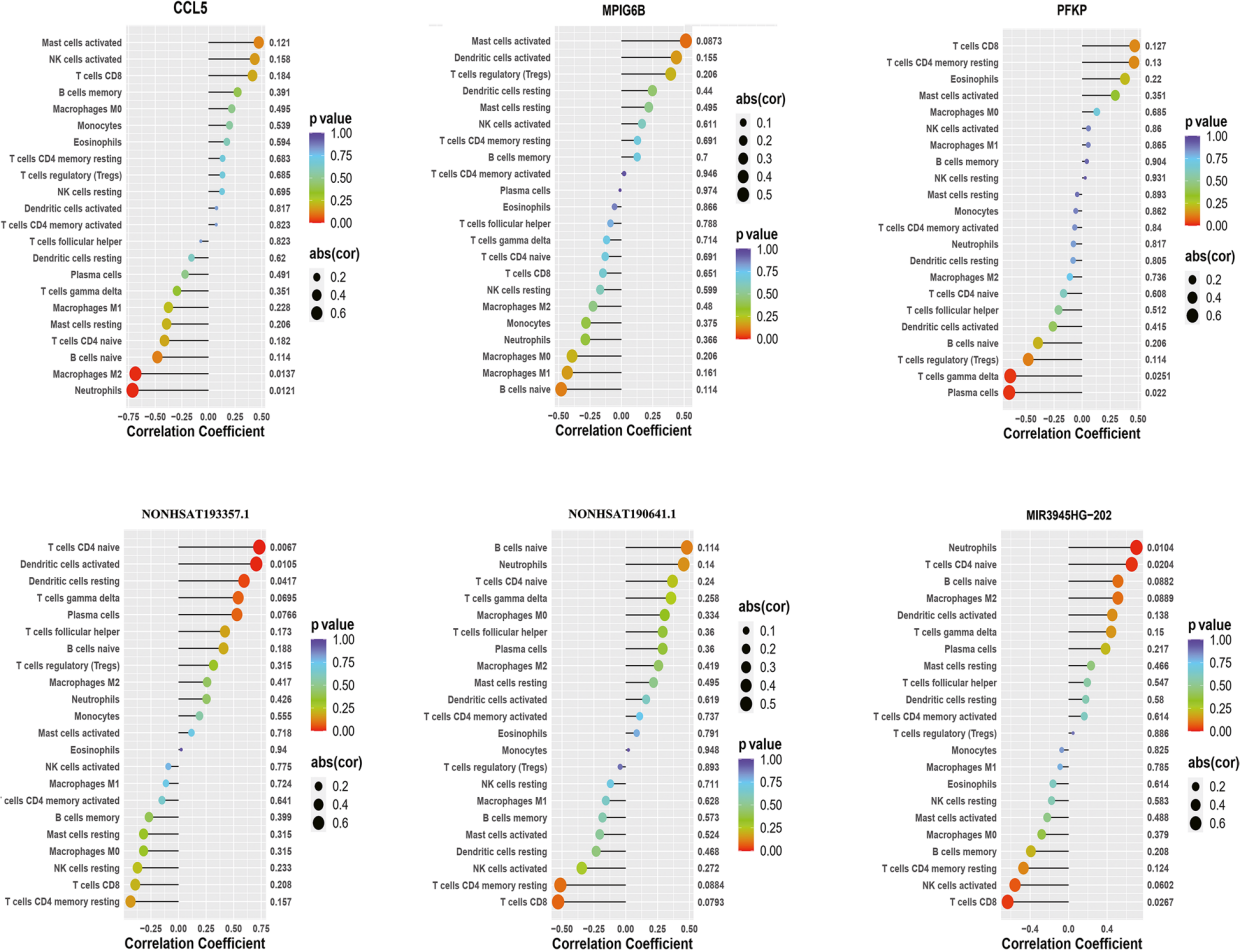
Correlation between immune cells and DE-lncRNAs (MIR3945HG-202, NONHSAT193357.1, and NONHSAT190641.1) or DE-mRNAs (CCL5, PFKP, and MPIG6B). R package “corrplot” was performed to analyse the correlation between infiltrating immune cells and DE-lncRNAs or DE-mRNAs. The colours of the dots represent the p-values DE-lncRNAs, differentially expressed lncRNAs; DE-mRNAs, differentially expressed mRNAs

Functional enrichment analysis of DE-mRNAs from serum exosomes in patients with RA showed significant enrichment in the immune response. Therefore, we used the CIBERSORT algorithm to predict the differences in immune cell infiltration among patients with RA, healthy controls, and patients with OA. The percentages of each of the 22 types of immune cells in each sample were shown in the bar plot (Fig. 6A). The scoring matrix of different cell types in each sample was shown in the heat map (Fig. 6B). The vioplot of the difference in immune cell infiltration reveal that patients with RA had a higher level of neutrophils than healthy controls and patients with OA (Fig. 6C). Correlation analysis between immune cells and several DE-lncRNAs and DE-mRNAs was performed. As illustrated in Fig. 7, CCL5 expression was negatively correlated with M2 macrophages and neutrophils. PFKP levels were negatively correlated with gamma delta T cells and plasma cells. NONHSAT193357.1 was positively associated with naïve CD4^+^ T cells, activated dendritic cells, and resting dendritic cells. MIR3945HG-202 was positively associated with neutrophils and naïve CD4^+^ T cells while being negatively related with CD8^+^ T cells.

## Discussion

RA is a chronic, systemic autoimmune disease characterised by joint inflammation and the progressive destruction of cartilage and bone. Accumulating studies have demonstrated that exosomes play a pivotal role in immune regulation and have been implicated in the pathogenesis of various autoimmune diseases [20, 21]. To the best of our knowledge, this is the first study to comprehensively describe the expression, biological functions, and potential clinical applications of DE-lncRNAs and DE-mRNAs in serum exosomes of patients with RA.

In this study, we found that CCL5-mRNA and MPIG6B-mRNAexpression levels were reduced in the serum exosomes of patients with RA compared to those of patients with OA, which was consistent with the RNA-sequencing results. As a member of the CC chemokine family, CCL5 is expressed in different cell types, such as fibroblasts, endothelial cells and mesangial cells [22–24]. CCL5 has strong chemotactic activity towards multiple immune cells, including eosinophils, astrocytes, mast cells, monocytes, CD4^+^ T cells, and CD8^+^ T cells [25–28]. Furthermore, CCL5 is associated with inflammatory arthritis, as it promotes leukocyte activation and recruitment to inflammatory sites [29]. MPIG6B, also known as G6b-B, is a type I transmembrane glycoprotein that is primarily expressed in platelets and acts as a critical regulator of platelet production, aggregation, and activation [30, 31]. G6b-B loss-of-function or G6b-B knockout mice develop aberrations in platelet production and activation [32, 33]. Platelets regulate leukocyte recruitment by releasing numerous inflammatory mediators [34]. We previously found that blood platelet count was significantly associated with RA disease activity [35]. In this study, we found that patients with RA presented a decreasing trend in the inflammation-associated indices CCL5-mRNA and MPIG6B-mRNA in serum exosomes compared to patients with OA, suggesting that there may be negative feedback in the transcription level of CCL5-mRNA and MPIG6B-mRNA derived from serum exosomes in patients with RA.

Functional and pathway enrichment analysis showed that the common functions of DE-lncRNAs and DE-mRNAs in RA were mainly immune-related pathways, indicating that DE-lncRNAs and DE-mRNAs in the serum exosomes of patients with RA are likely to be critical factors in RA pathogenesis and may also affect the host immune response. A previous transcriptomic analysis showed the increased expression of several chemokines in the synovial fluid of patients with RA [36]. To understand the role of infiltrating immune cells, we performed CIBERSORT analysis to investigate the distribution of immune cell infiltration in patients with RA according to the DE-mRNAs in serum exosomes. We found that neutrophil levels were higher in patients with RA than in healthy controls or patients with OA. Neutrophils are the most abundant cell type in RA synovial fluid [37] and can also be detected in RA synovial tissues [38, 39]. The pathogenic role of neutrophils in RA is related to several processes, including increased migratory capacity and cell survival, enhanced inflammatory activity, and exacerbated release of neutrophil extracellular traps [40]. Our findings suggest that serum exosome mRNAs from neutrophils is involved in RA pathogenesis.

GSEA showed that the most significant gene sets enriched in RA were associated with the downregulation of metabolism-related terms, such as oxidative phosphorylation, glycolysis, and amino acid biosynthesis. Our findings were consistent with the results of a previous study showing that naïve CD4^+^ T cells isolated from patients with RA exhibit diminished glycolytic activity [41]. Low ATP production by circulating CD4^+^ T cells was detected in patients with RA in a Japanese cohort; furthermore, mitochondrial failure may result in lower ATP generation and reduced ROS release [42, 43]. Moreover, another study reported that phosphofructokinase PFKFB3 deficiency could impair ATP generation, autophagy, and redox balance in the T cells of patients with RA [41]. Indeed, given the contribution of DE-mRNAs in serum exosomes to the metabolic regulation of RA, more comprehensive analyses of the effects of these DE-mRNAs on immunometabolism and their underlying mechanisms in RA may lead to a better understanding of RA pathogenesis.

Exploration of the regulatory interactions between DE-lncRNA and target mRNAs is critical for elucidating lncRNA-mediated gene regulation in RA, as emerging data show that lncRNA regulation is involved in the innate and adaptive immune responses [44, 45]. In this study, we found that the enriched GO functions of lncRNAs specifically expressed in RA were related to cell proliferation, cellular metabolic processes, and cell communication. Similar to our findings, previous studies have reported that lncRNAs regulate glycolysis and glutaminolysis [46, 47]. The identification of DE-lncRNAs that directly target mRNAs in RA will help uncover novel regulatory mechanisms of serum exosomes. However, further functional studies are required to validate the interactions between the DE-lncRNA and target mRNAs identified in this study.

## Conclusion

In conclusion, our results demonstrate that the functions of DE-lncRNAs and DE-mRNAs identified in serum exosomes of patients with RA are associated with immune response and metabolic regulation. Patients with RA presented with decreased levels of CCL5-mRNA and MPIG6B-mRNA in serum exosomes compared to patients with OA. Increased neutrophil infiltration may be a potential mechanism underlying RA pathogenesis. However, this study has several limitations. First, considering the limited number of samples, further validation in future studies is required. Secondly, the biological functions of DE-lncRNAs and DE-mRNAs in RA pathogenesis should be explored experimentally. Moreover, the association between DE-mRNAs in serum exosomes and neutrophils should be studied further.

### Supplementary Information

**Additional file 1: Fig. S1.** Gene Set Enrichment Analysis (GSEA) of differentially expressed mRNAs in serum exosomes of RA patients. A-B: Biological process term (A) and KEGG pathway enrichment (B) of the differentially expressed mRNAs in RA patients compared with healthy controls. C-D: Biological process term (C) and KEGG pathway enrichment (D) of the differentially expressed mRNAs in RA patients compared with OA patients. KEGG: Kyoto Encyclopedia of Gene and Genome. RA, Rheumatoid arthritis; OA, Osteoarthritis.

**Additional file 2: Fig. S2.** Protein-protein interaction (PPI) network of differentially expressed mRNAs in serum exosomes of RA patients compared with OA patients. Three clusters were generated with 28 genes in cluster 1 (red bubbles), 28 genes in cluster 2 (green bubbles) and 21 genes in cluster 3 (blue bubbles). The annotations of functional enrichment of genes in cluster 2 is shown. The functional enrichment of genes in cluster 1 and cluster 3 is not significant. RA, Rheumatoid arthritis; OA, Osteoarthritis.

**Additional file 3: Fig. S3.** Primary images of western blotting for the detection of proteins of exosomes isolated from human serum (supporting images for Fig. 1C).

## Data Availability

All data that support the findings of this study are available from the corresponding author upon reasonable request. RNA sequencing data are available in the BioProject database of NCBI (project accession number PRJNA911001).

## Abbreviations

RA: Rheumatoid arthritis
RF: Rheumatoid factor
Anti-CCP: Anti-cyclic citrullinated peptide
EVs: Extracellular vesicles
MiRNAs: MicroRNAs
LncRNAs: Long non-coding RNAs
PBMCs: Peripheral blood mononuclear cells
DE-lncRNAs: Differentially expressed lncRNAs
DE-mRNAs: Differentially expressed mRNAs
OA: Osteoarthritis
NTA: Nanoparticle tracking analysis
TEM: Transmission electron microscopy
GO: Gene Ontology
KEGG: Kyoto Encyclopedia of Genes and Genomes
GSEA: Gene set enrichment analysis
PPI: Protein-protein interaction
PCA: Principal component analysis
ROC: Receiver operating characteristic
AUC: Area under the ROC curve
