# Supplementary figures and images for: Comprehensive analysis of serum exosome-derived lncRNAs and mRNAs from patients with rheumatoid arthritis
Source: Arthritis Res Ther. 2023 Oct 16;25:201. doi: 10.1186/s13075-023-03174-9 (PMC10577909; doi:10.1186/s13075-023-03174-9)

A

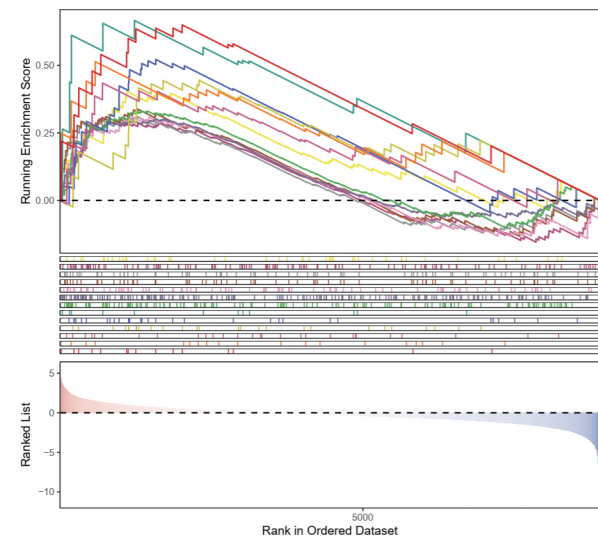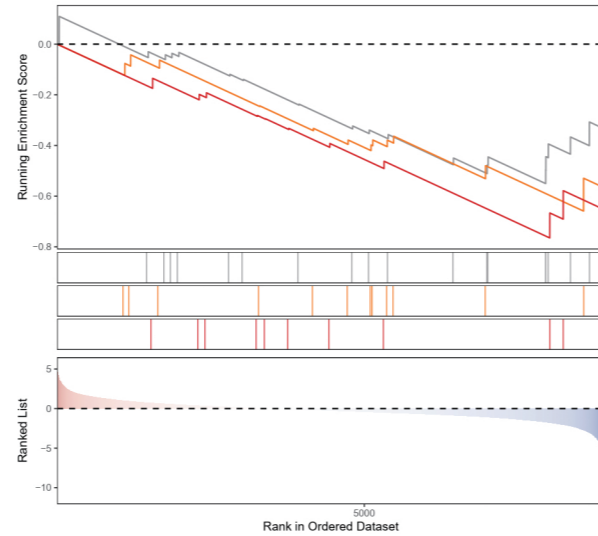

B

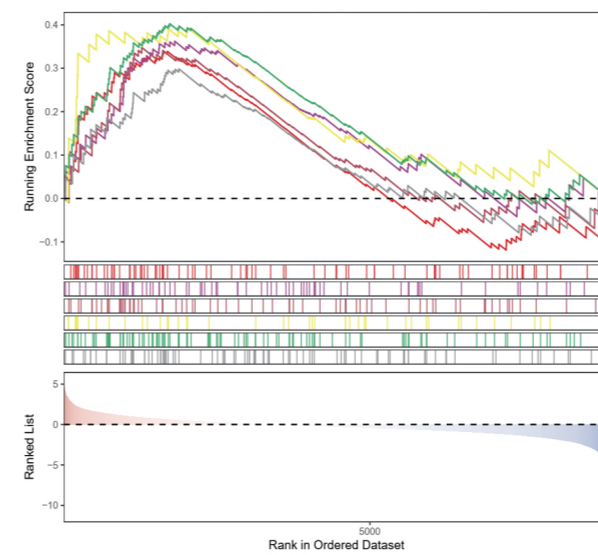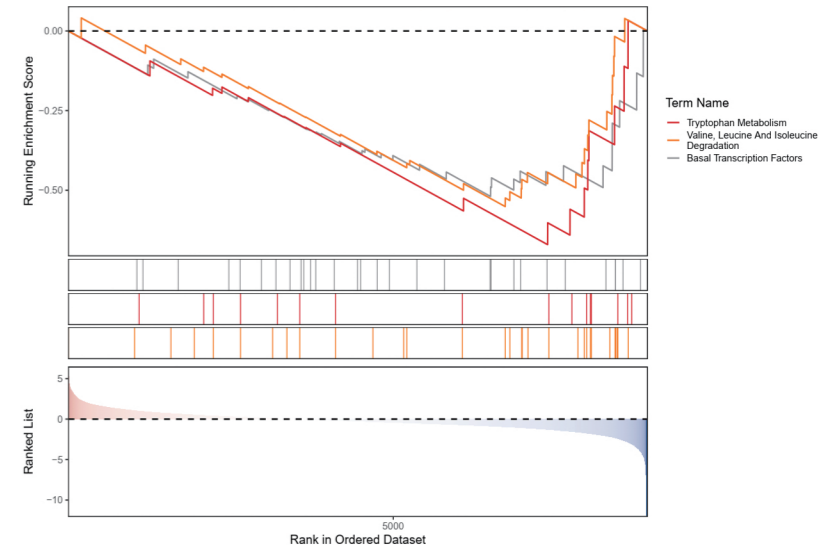

C

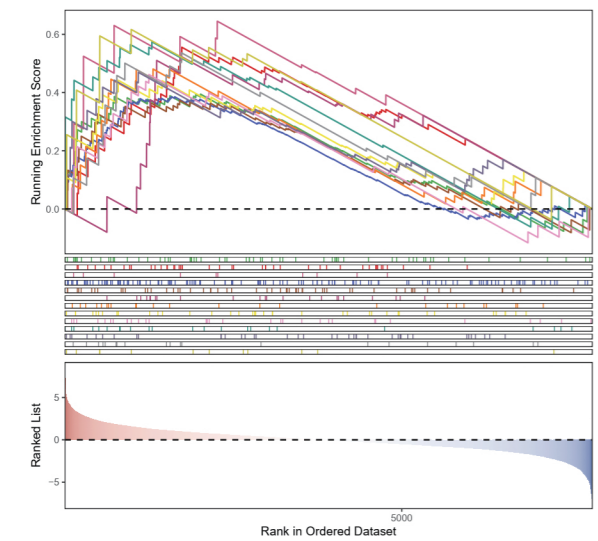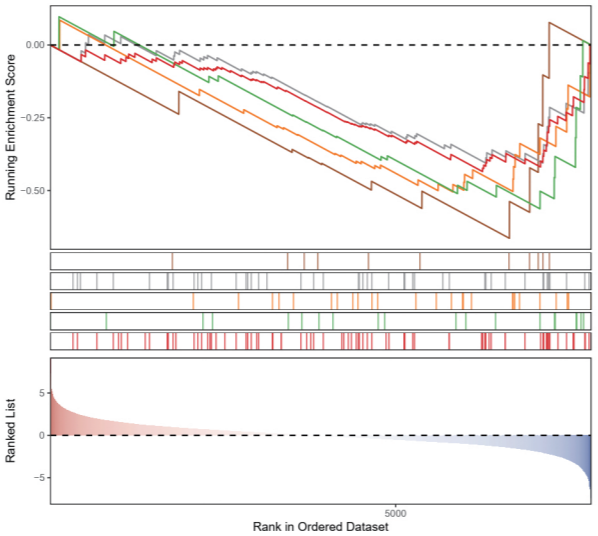

D

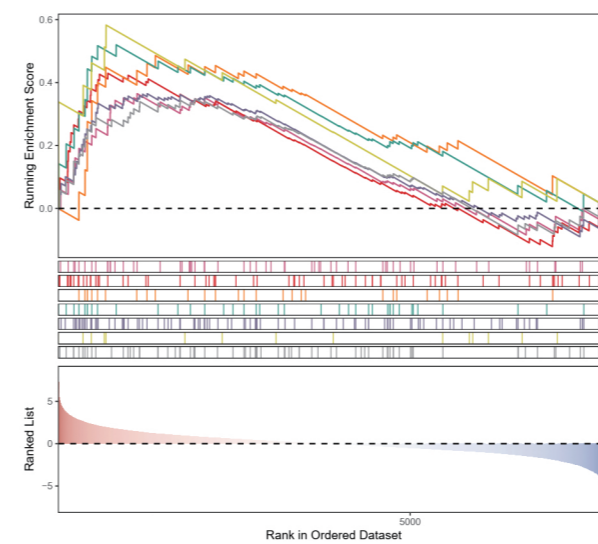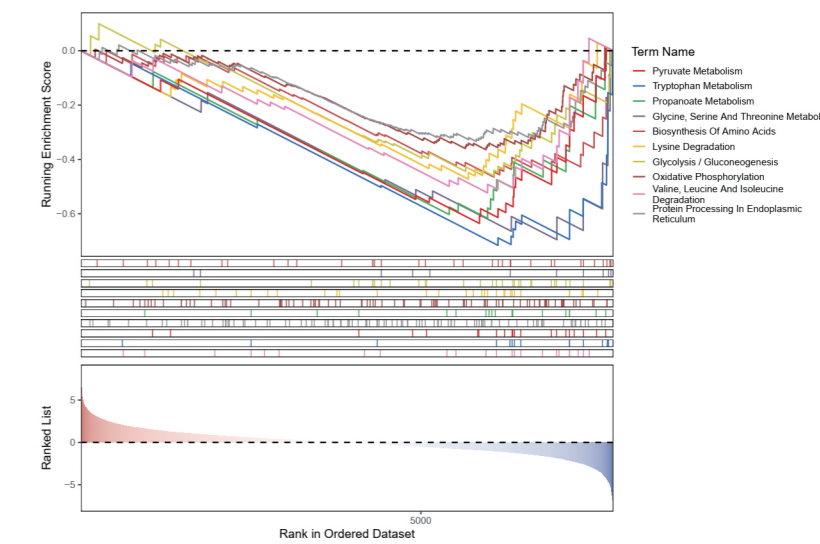

Supplement: Supplementary file 1 — Additional file 1: Fig. S1. Gene Set Enrichment Analysis (GSEA) of differentially expressed mRNAs in serum exosomes of RA patients. A-B: Biological process term (A) and KEGG pathway enrichment (B) of the differentially expressed mRNAs in RA patients compared with healthy controls. C-D: Biological process term (C) and KEGG pathway enrichment (D) of the differentially expressed mRNAs in RA patients compared with OA patients. KEGG: Kyoto Encyclopedia of Gene and Genome. RA, Rheumatoid arthritis; OA, Osteoarthritis. [file 13075_2023_3174_MOESM1_ESM.pdf]

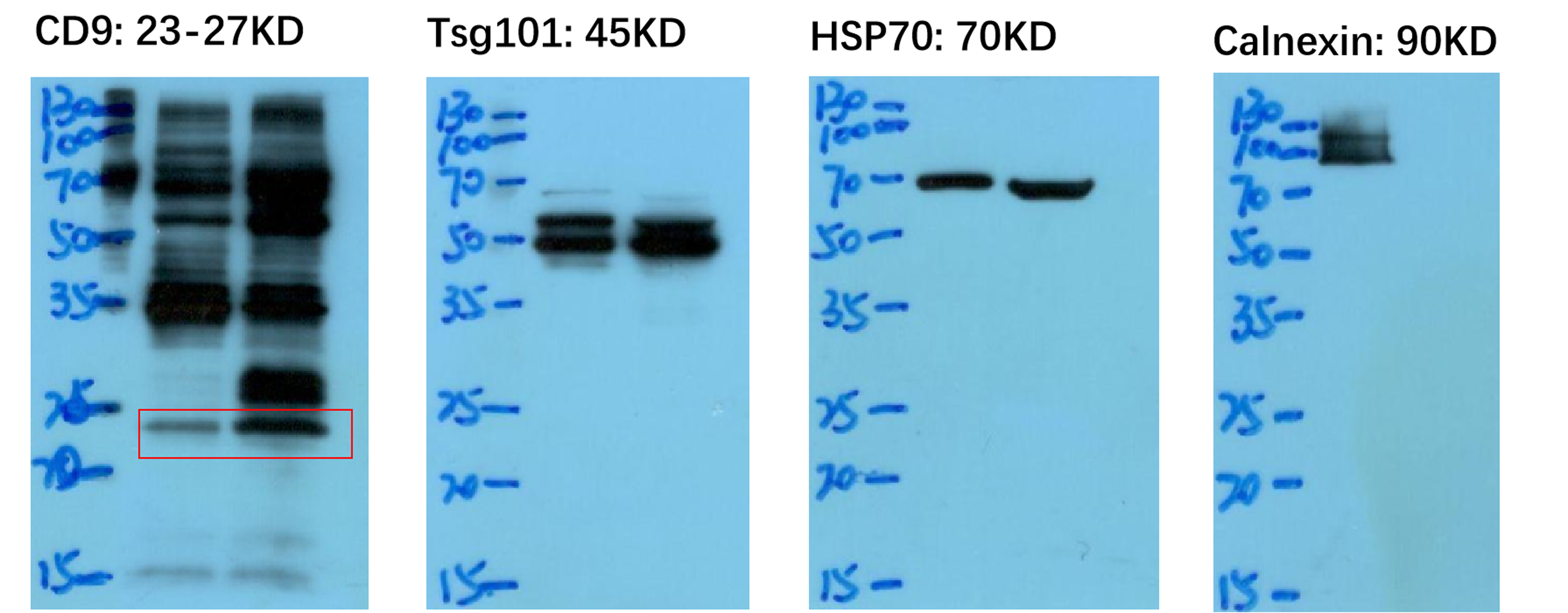

Supplement: Supplementary file 3 — Additional file 3: Fig. S3. Primary images of western blotting for the detection of proteins of exosomes isolated from human serum (supporting images for Fig. 1C). [file 13075_2023_3174_MOESM3_ESM.tif]
