# Supplementary material for: Comprehensive analysis of serum exosome-derived lncRNAs and mRNAs from patients with rheumatoid arthritis
Source: Arthritis Res Ther. 2023 Oct 16;25:201. doi: 10.1186/s13075-023-03174-9 (PMC10577909; doi:10.1186/s13075-023-03174-9)
Supplement: Supplementary file 2 — Additional file 2: Fig. S2. Protein-protein interaction (PPI) network of differentially expressed mRNAs in serum exosomes of RA patients compared with OA patients. Three clusters were generated with 28 genes in cluster 1 (red bubbles), 28 genes in cluster 2 (green bubbles) and 21 genes in cluster 3 (blue bubbles). The annotations of functional enrichment of genes in cluster 2 is shown. The functional enrichment of genes in cluster 1 and cluster 3 is not significant. RA, Rheumatoid arthritis; OA, Osteoarthritis. [file 13075_2023_3174_MOESM2_ESM.pdf]

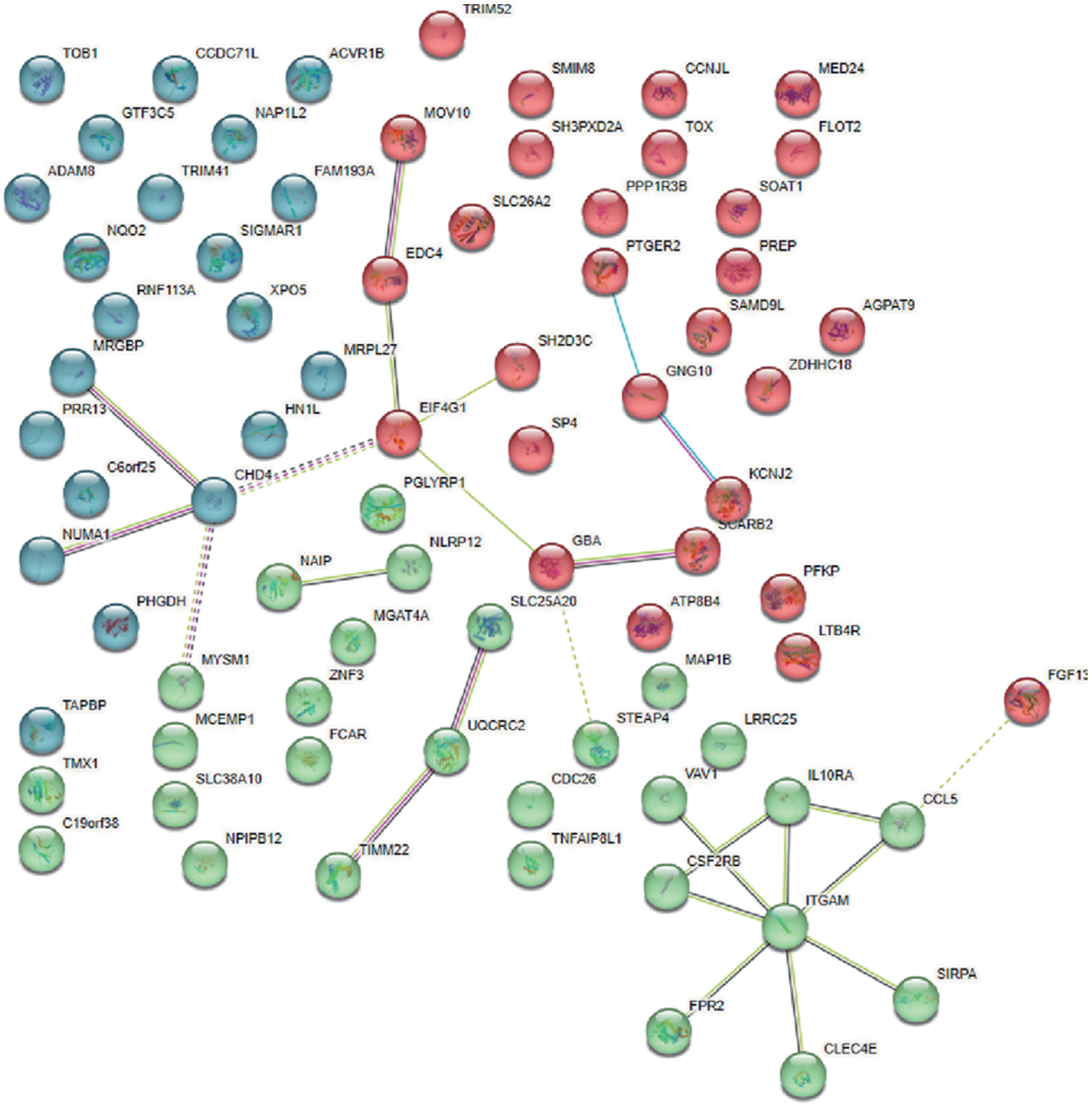

CLUSTER 2: 28 genes

PPI enrichment p-value: 0.000168

Biological processes:

Leukocyte activation (FDR = 0.0023)

Neutrophil activation (FDR = 0.017)

Innate immune response (FDR = 0.0379)

Cellular response to cytokine stimulus (FDR = 0.0387)

Leukocyte migration (FDR = 0.0406)
